# Boron and Nitrogen Co-Doped Porous Graphene Nanostructures for the Electrochemical Detection of Poisonous Heavy Metal Ions

**DOI:** 10.3390/nano14090806

**Published:** 2024-05-06

**Authors:** Yogesh Chaudhary, Shradha Suman, Benadict Rakesh, Gunendra Prasad Ojha, Uday Deshpande, Bishweshwar Pant, Kamatchi Jothiramalingam Sankaran

**Affiliations:** 1 CSIR-Institute of Minerals and Materials Technology, Bhubaneswar 751013, India; yogesh.immt23a@acsir.res.in (Y.C.); shradha.2021@immt.res.in (S.S.); benadict@immt.res.in (B.R.); 2 Academy of Scientific and Innovative Research (AcSIR), Ghaziabad 201002, India; 3 Carbon Composite Energy Nanomaterials Research Center, Woosuk University, Wanju 55338, Republic of Korea; gpojha10@gmail.com; 4 UGC-DAE Consortium for Scientific Research, Khandwa Road, Indore 452001, India; uday@csr.res.in

**Keywords:** laser-induced graphene, co-doping, electrochemical sensor, square wave voltammetry, heavy metal ions

## Abstract

Heavy metal poisoning has a life-threatening impact on the human body to aquatic ecosystems. This necessitates designing a convenient green methodology for the fabrication of an electrochemical sensor that can detect heavy metal ions efficiently. In this study, boron (B) and nitrogen (N) co-doped laser-induced porous graphene (LIG_BN_) nanostructured electrodes were fabricated using a direct laser writing technique. The fabricated electrodes were utilised for the individual and simultaneous electrochemical detection of lead (Pb^2+^) and cadmium (Cd^2+^) ions using a square wave voltammetry technique (SWV). The synergistic effect of B and N co-doping results in an improved sensing performance of the electrode with better sensitivity of 0.725 µA/µM for Pb^2+^ and 0.661 µA/µM for Cd^2+^ ions, respectively. Moreover, the sensing electrode shows a low limit of detection of 0.21 µM and 0.25 µM for Pb^2+^ and Cd^2+^ ions, with wide linear ranges from 8.0 to 80 µM for Pb^2+^ and Cd^2+^ ions and high linearity of R^2^ = 0.99 in case of simultaneous detection. This rapid and facile method of fabricating heteroatom-doped porous graphene opens a new avenue in electrochemical sensing studies to detect various hazardous metal ions.

## 1. Introduction

Competitive industrialisation and overexploitation of natural resources have polluted water resources enormously [1]. Heavy metals (HMs), the toxic elements present in the improper disposal of untreated water from refineries and mining factories create major environmental issues for humankind and aquatic ecosystems [2]. In general, HMs have a high atomic mass and a density greater than 5 g/cc, such as lead (Pb), cadmium (Cd), arsenic (As), mercury (Hg), chromium (Cr), etc. [3]. Among these HMs, Pb and Cd belong to the top lethargic HMs, which have life-threatening toxicity on long exposure to our body and are also non-biodegradable [4]. Pb toxicity causes adversities such as miscarriages, renal dysfunction, injury to peripheral nerves, and cognitive dysfunction [4,5]. Similarly, Cd poisoning causes nephrotoxicity, central nervous system complications, osteoporosis, and even cancer [6]. Considering these fatal complications, these HMs need to be monitored qualitatively as well as quantitatively. Therefore, a rapid, selective, sensitive, analytical technique is required. Conventional monitoring techniques such as atomic absorption spectroscopy [7], inductively coupled plasma mass spectrometry [8], and atomic fluorescence spectroscopy [9] to detect HMs are accurate and reliable. However, these techniques are expensive and time-consuming. Also, the requirement of trained expertise makes them inconvenient for practical and onsite use. Thus, it necessitates a search for a cost-effective, portable, and highly responsive technique for onsite monitoring [10], for which the electrochemical sensing technique is the suitable one.

Square wave voltammetry (SWV), one of the electrochemical techniques, is highly sensitive, has a quick response, low faradic current, and is also useful for trace analysis with low detection of limits [11]. In the electrochemical setup, the working electrode is one of the important components of an electrochemical sensor, as a sensing material. Therefore, for suitable sensing material for an electrochemical sensor, graphene and its derived materials are better options because of their high specific surface area, excellent electrical conductivity, and electro-catalytic activity in several redox processes [12]. Also, the nanostructuring increases the aspect ratio, which increases the active sites for sensing HMs. Graphene-based electrodes have become a robust player in the field of electrochemical sensing such as heavy metals [13], pesticides [14], and other water contaminants [15,16].

However, various conventional routes for synthesising graphenes such as chemical vapour deposition, thermal annealing, wet chemical method, solvothermal, arc discharge, and lithography entail lengthy procedures, high-temperature treatment, and hazardous experimental conditions, which limit their widespread practical applications [15]. Thus, it necessitates finding a novel route, which can be cheaper, rapid, and applicable to scientific research, especially in the electrochemical sensing arena. Recently, direct laser writing (DLW) has been a scalable, flexible, and eco-friendly fabrication method for producing graphene on flexible substrates [17]. The obtained laser-induced graphene (LIG) has been used as a working electrode for various electrochemical applications such as supercapacitors [18], sensors [19], and electrocatalysis [20]. Xueni et. al. developed a modification of the glassy carbon electrode (GCE) using synthesised nitrogen-doped laser-engraved graphene (N@LEG) by introducing polyaniline (PANI) and polyvinylpyrrolidone (PVP) as N-dopant. The N@LEG modified glassy carbon electrode (N@LEG/GCE) with in situ bismuth film modification showed enhanced electrochemical properties and a 3D porous structure with large electrochemical active surface areas. This combination, along with the strong metal ion affinity of nitrogen atoms, significantly improved the detection range for heavy metals like Cd (II) and Pb (II). The sensor was successfully utilised for the simultaneous determination of Cd (II) and Pb (II) using square wave anodic stripping voltammetry (SWASV) with optimised conditions [21]. Jeong et al. developed an electrode using silver nanoparticles and laser-induced graphene (AgNP/LIG) for the individual and simultaneous detection of the cadmium (Cd), lead (Pb), and copper (Cu) ions using the square wave anodic stripping voltammetry (SWASV) method with optimal conditions [22]. Saisree et al. studied the effects of modifying GCE, with sulphur co-doped nitrogen graphene quantum dots (S, N-GQD) (4 nm), prepared by hydrothermal reaction using polyaniline and sulphric acid as an acid catalyst and S-doping agent. The authors used the modified electrode to detect individually and simultaneously Cd (II), Pb (II), and Hg (II) by DPV technique [23]. However, the fabrication of graphene involved lengthy procedures and additional layers of polymers, which complicated the process. However, the application of pristine LIG is restricted due to its absence of an intrinsic bandgap [24], and, thus, needs modification for its effective and result-oriented usage.

To open up the bandgap, several strategies have been adopted such as molecular adsorption, chemical functionalisation, doping of heteroatoms, and edge-effects-induced bandgap [25]. Also, heteroatom-doped graphene has piqued the interest of researchers due to its superior physicochemical, electromagnetic, and structural capabilities as compared to undoped graphene [26]. Several doping elements were successfully doped in LIG [27,28,29]. Tour et al. [24] synthesised boron-doped LIG (LIG_B_) by using H_3_BO_3_ and polyamic acid (PAA) solution to form PI/H_3_BO_3_ film, which was finally transformed into LIG_B_ using CO_2_ laser irradiation. Han et al. [28] demonstrated the in situ fabrication of nitrogen-doped LIG (LIG_N_) using a composite of melamine (C_3_H_6_N_6_) and PAA as the N precursor and converted into LIG_N_ by using laser pyrolysis methodology. Khandelwal et al. [29] reported a duplicate laser pyrolysis method for fabricating B and N co-doped LIG (LIG_BN_) for electrochemical supercapacitor applications. However, the procedure of fabrication of LIG involving precursor H_3_BO_3_ and PAA solution, additional layer formation, and thermal treatment made the process lengthy, expensive, and tedious.

Based on the above understanding, herein, a rapid, green, and simple methodology to dope heteroatoms such as boron and nitrogen using a DLW technique was developed in which, boric acid and urea, both affordable and ecologically benign, were utilised as boron and nitrogen precursors, respectively, to enhance defects and improve catalytic activity. The synthesised samples are characterised using microscopic and spectroscopic techniques to study the doping effects. Aiming for the application LIG_BN_, the electrochemical sensing for toxic heavy metal ions such as Pb^2+^ and Cd^2+^ was performed with an appreciable limit of detection and sensitivity. This work presents a new arena for the electrochemical detection of HMs, using a heteroatom-doped LIG by DLW technique, which reduces the number of fabrication steps.

## 2. Materials and Methods

### 2.1. Materials and Reagents

Commercial polyimide (PI) sheets of a thickness of 0.125 mm were purchased from Cole Parmer, Cat. No. 08277-88, India. Potassium ferricyanide (K_3_[Fe(CN)_6_]), 99.0% pure was from Himedia Laboratories Pvt. Ltd., Kolkata, India; potassium ferrocyanide (K_4_Fe(CN)_6_·3H_2_O), 99.0% pure was from S.D. Fine Chemical Limited, Mumbai, India; potassium chloride (KCl), sodium acetate (CH_3_COONa, NaAc), acetic acid (CH_3_COOH, HAc), boric acid (H_3_BO_3_), and urea (NH_2_CONH_2_), 99.5% pure was purchased from Thermo Fischer Scientific Pvt. Ltd., Hyderabad, India.

Lead nitrate (Pb(NO_3_)_2_, 99% pure from Sisco Research Laboratories Pvt. Ltd., Mumbai, India, and cadmium chloride (CdCl_2_), 95% pure from Qualigens Fine Chemicals Pvt. Ltd., Bangalore, India, were bought to perform experiments. All chemicals were of analytical grade. All of these chemicals were used as such without further purification. An amount of 0.1 M NaAc/HAc buffer of different pH was prepared by mixing a suitable amount of NaAc and HAc.

Ultra-pure water from a Milli-Q^®^ Direct Water Purification System (resistivity 18.2 MΩ·cm at 25 °C), Merck Life Science Pvt. Ltd., Bangalore, India, was used throughout the experiments.

### 2.2. Fabrication of Boron and Nitrogen Co-Doped Laser-Induced Graphene

**Figure 1:**
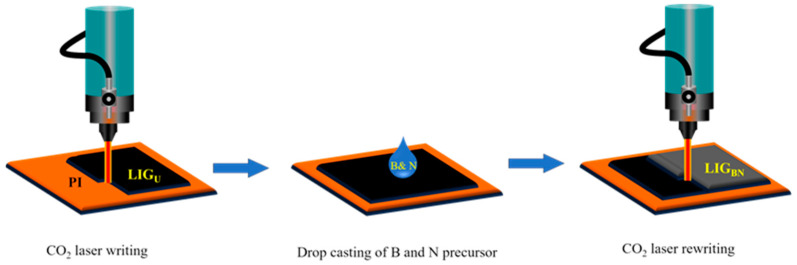
Schematic diagram for the fabrication of LIG_BN_ using CO_2_-laser assisted method.

Figure 1 illustrates the processing steps of fabrication of LIG_BN_ electrodes. The PI sheets were cleaned by rinsing them with ethanol and then dried. Then, the first DLW was executed using a CO_2_-based laser-engraver machine, Meera Lasers Solution Pvt. Ltd., Chennai, India, on PI (1 cm × 1 cm) sheets at a laser power of 50% of 30 W and a scanning speed of 300 mm/s in ambient conditions to form graphene on PI. The lasing parameters can impact the structure and morphology of the LIG, which, in turn, may affect its surface area, porosity, and functional groups. These factors can influence the adsorption capacity and kinetics of heavy metal ions on the LIG surface. However, the impact of lasing parameters on the quality of LIG would require experimental investigation and optimisation of the laser system, which is carried out by our group [30]. The laser parameters, such as scan speed (200–300 mm/s) and laser power (40–55%), were varied to optimise the process. The laser head and substrate were separated by 10 mm for each experiment. A 0.05 mm interval was maintained between two consecutive laser passing lines when using the Y-unilateral scanning mode. Thus, the lasing parameters such as scan speed and laser power were optimised, which can indirectly influence the sensor response by affecting the properties of LIG. The optimised parameters were 300 mm/s scanning speed and 50% for LIG for the high degree of graphitisation. Thus, the obtained LIG is termed an undoped LIG (LIG_U_). Since LIG_U_ behaves hydrophobic [31], it was treated under home-built dielectric barrier discharge plasma at 14 kV for 2 min, which made the LIG_U_ hydrophilic. Then, LIG_BN_ was fabricated by drop casting of 50 µL of 1% by mass of aqueous H_3_BO_3_ and NH_2_CONH_2_ on LIG_U_, and the sample was dried at 70 °C for 30 min. Next, the second laser rewriting was carried out with the same laser parameters to incorporate B and N into the graphene lattice due to the reorganisation and restructuring of graphene nanostructures. In this way, undoped LIG (LIG_U_), LIG_B_, and LIG_N_ were also fabricated for comparison.

### 2.3. Characterisation

The morphology and microstructure of LIG_BN_ were examined using field emission scanning electron microscopy (FE-SEM; JSM-IT 800, JEOL, Tokyo, Japan) and high-resolution transmission electron microscopy (HR-TEM; JEM-F200 200 kV, JEOL, Tokyo, Japan). The bonding characteristics, defects, and chemical states of LIG_BN_ were analysed using a Raman spectrometer, Renishaw in Via with a 532 nm laser, Renishaw Metrology system Pvt. Ltd., Chennai, India) and X-ray photoelectron spectroscopy, SPECS scientific instruments Inc., Berlin, Germany. The ImpedanceEdge^®^ Multiparameter pH Meter, 230 V SKU: HI2020-02, Hanna Instruments Pvt. Ltd., Mumbai, India, was used to determine the pH values of the buffer.

### 2.4. Electrochemical Measurements

Electrochemical measurements were performed on a portable PalmSens (EmStat 4LR), the Netherlands, electrochemical workstation with a conventional three-electrode system consisting of a doped LIG as working electrode, an Ag/AgCl saturated KCl reference electrode, and a platinum wire counter electrode. The CVs were implemented with the potential varied from −0.2 V to 0.8 V at a scan rate of 50 mV s^−1^. The electrochemical impedance spectroscopy (EIS) measurements were carried out with the frequency from 0.1 Hz to 100 kHz. Both of them were run in 5.0 mM [Fe (CN)_6_]^3−/4−^, a solution containing 0.1 M KCl. Furthermore, SWV was employed for the individual and simultaneous detection of Cd^2+^ and Pb^2+^ with a step potential at 5 mV and a scan potential from −1.0 V to −0.2 V with 250 mV amplitude, 1 Hz frequency, and in 0.1 M acetate (NaAc/HAc) buffer (pH = 4.5).

## 3. Results and Discussions

### 3.1. Morphological and Microstructural Studies

**Figure 2:**
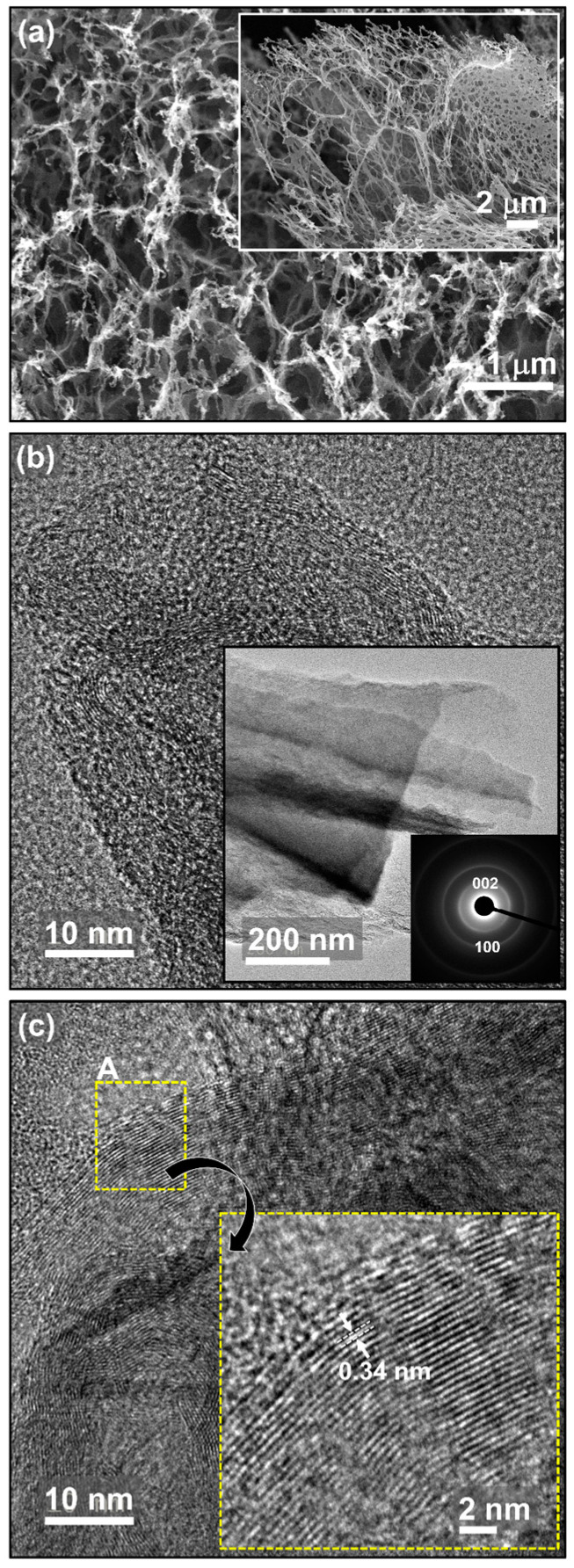
(**a**) FESEM micrograph of porous LIG_BN_ @ 1 µm scale. The inset of (**a**) shows the low-magnified FESEM micrograph; (**b**,**c**) HR-TEM micrographs of LIG_BN_ @ 10 nm scale. The inset of (**b**) shows the low-magnified TEM micrograph along with its corresponding SAED pattern. The inset of (**c**) shows the high-magnified HRTEM micrograph of region A of (**c**).

The surface morphologies of LIG_BN_ were characterised by FESEM. Figure 2a shows the formation of an interlinked porous network of graphene structures, which might be due to the localised and on-spot high temperature of laser beams on PI. The PI decomposes into gaseous products so that the entrapped gases may create porous structures during the graphitisation process [32]. LIG_BN_ maintains the porous structure, displaying the pore size in the range of 0.8–1.7 µm (inset of Figure 2a). The microstructure of LIG_BN_ was analysed by HRTEM. The low-magnified TEM image of LIG_BN_ at the inset of Figure 2b shows wrinkled structures of graphene. The diffraction rings signifying (100) and (002) planes of graphene in the selective area diffraction pattern (inset of Figure 2b) show the polycrystalline structure of LIG_BN_. Moreover, the HRTEM images shown in Figure 2b,c indicate that the LIG_BN_ possesses few-layered features of graphene nanostructures. The high-magnified HRTEM image corresponds to region A of Figure 2c (inset of Figure 2c) representing the clear fringes with a characteristic d-spacing of 0.34 nm, corresponding to the (002) plane in graphitic materials. Consequently, the presence of the 3D porous interconnected graphene wrinkled structures provides such a large specific surface area and numerous reactive sites that make it easier to accumulate metal ions and contribute to the wide detection range [33].

### 3.2. Raman Analysis

**Figure 3:**
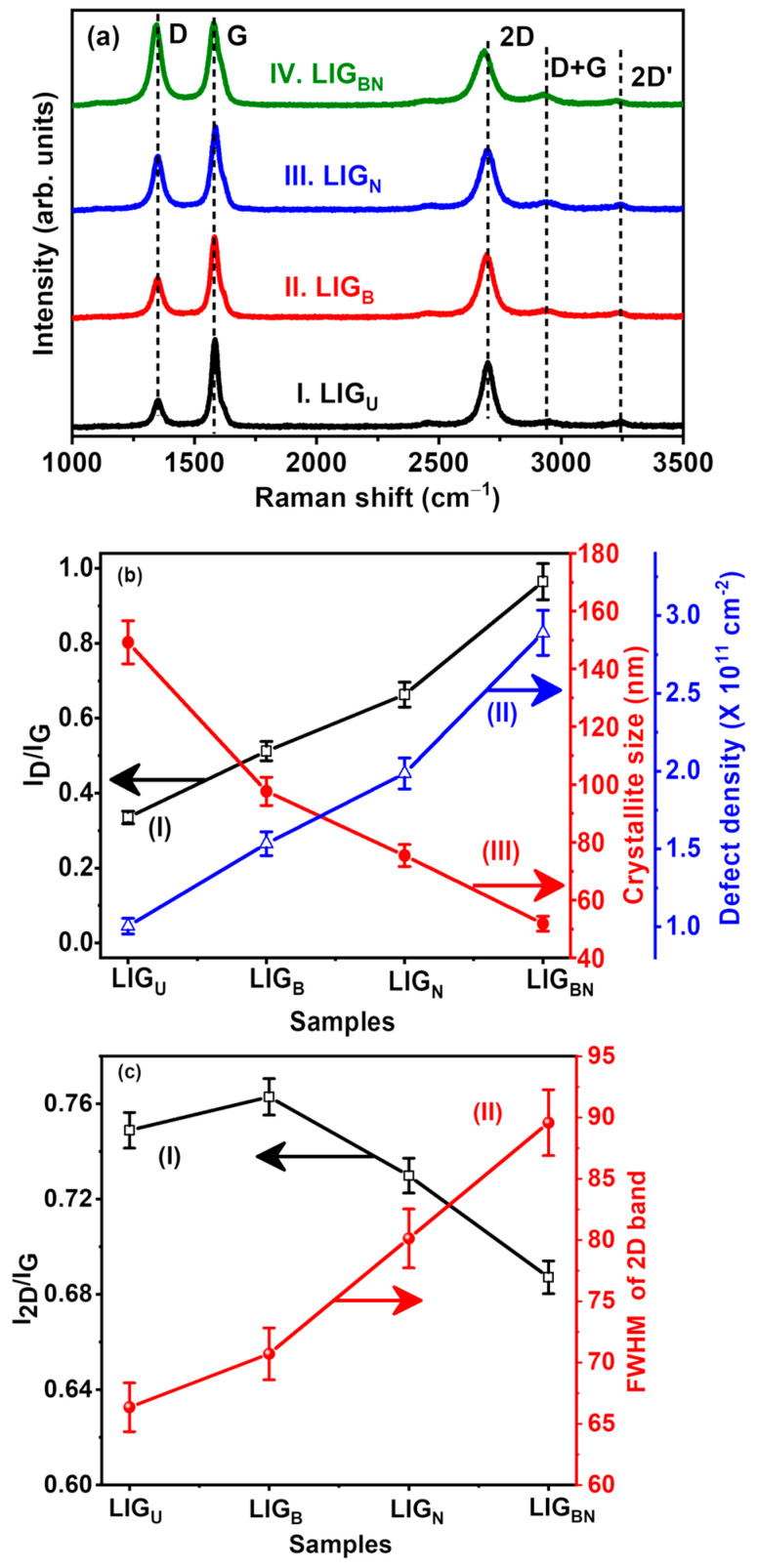
(**a**) Raman spectra of LIG samples (I) LIG_U_, (II) LIG_B_, (III) LIG_N_, and (IV) LIG_BN_. Variation in (**b**) I_D_/I_G_, crystallite size, and defect density of LIG samples, (**c**) I_2D_/I_G_ and FWHM of 2D band of LIG samples.

One of the powerful tools to characterise graphene and its derivatives is Raman spectroscopy [30], which was used for distinguishing the structural features of doped LIG samples. As exhibited in Figure 3a, the Raman spectra of the LIG samples showed three graphene indicative peaks, namely a D peak at approximately 1350 cm^−1^, a G peak at approximately 1580 cm^−1^, and a 2D peak at approximately 2700 cm^−1^. The G-peak is attributed to the first-order scattering of the E_2g_ optical mode in sp^2^ domains caused by carbon atom in-plane vibrations. The D-peak, on the other hand, is caused by disordered regions containing sp^3^ carbons associated with out-of-plane vibrations [21].

Additionally, the intensity ratio I_D_/I_G_, as indicated in Figure 3a, which is employed to evaluate the degree of defects and disorder in the sp^2^ hybridised graphitic carbon, is about 0.96 for LIG_BN_ and 0.32 for LIG_U_, revealing the crystal defect and disorder degree ascended after doping with boron and nitrogen [33]. Despite that, the distinct 2D peak further illustrates the generation of few-layered graphene [34], which is as per the FESEM results.

Furthermore, a detailed analysis of the crystallite size along the X-axis (La), defect density (nD), the distance between defects (L_D_), and the significance of I_D_/I_G_ and I_2D_/I_G_ of the LIG samples were carried out, particularly for LIG_U_, LIG_B_, LIG_N_, and LIG_BN_, respectively, as shown in Figure 3b,c, and listed in Table 1, respectively. It is observed in Figure 3c that the LIG_BN_ sample shows the full-width half maxima (FWHM) of 2D and I_2D_/I_G_ values of 89.56 cm^−1^ and 0.69, respectively. In Figure 3b, it is found that the LIG_BN_ sample has a low crystallite size (L_a_) of 51.84 nm, a smaller distance between defects (L_D_) of 14.12 nm, and a maximum defect density of 2.89 × 10^11^ cm^−1^ as compared to the other LIG samples, suggesting the formation of more defects [35]. Table 1 indicates the defect density increases on co-doping boron and nitrogen into LIG, which plays a key role in the creation of active sites for heavy metal ions adsorption.

### 3.3. XPS Analysis

**Figure 4:**
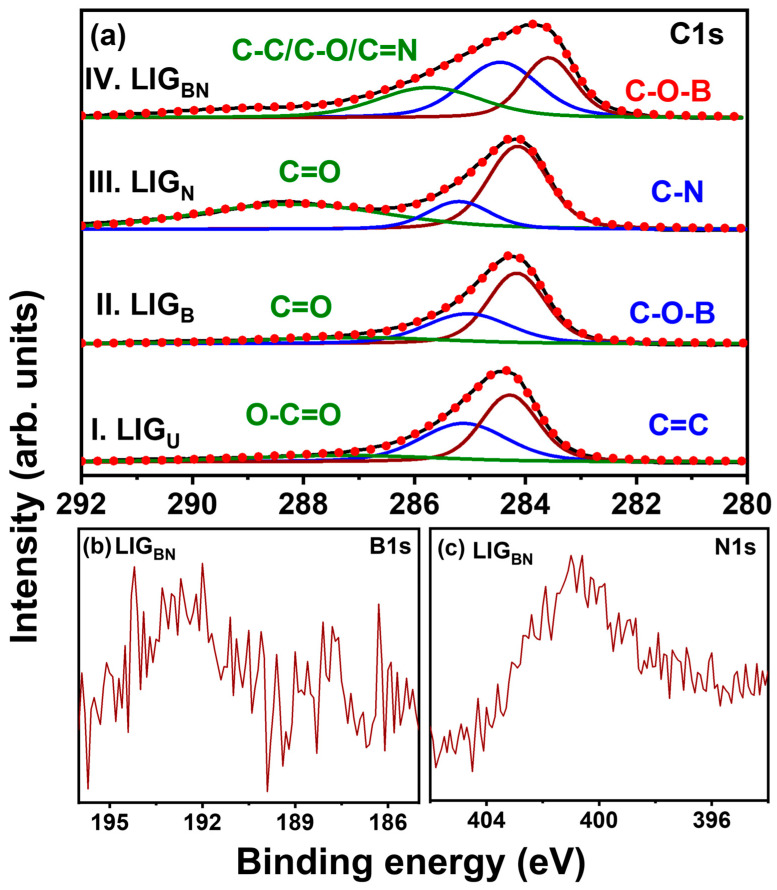
(**a**) C1s XPS spectra of LIG_U_, LIG_B_, LIG_N_, and LIG_BN_. (**b**) B1s and (**c**) N1s XPS spectra of LIG_BN_.

The XPS is an essential study to characterise the heteroatom doping into lattices of graphene and their bonding characteristics. Figure 4a depicts the C1s spectra of LIG_U_, LIG_B_, LIG_N,_ and LIG_BN_ in which the bonding configurations of carbon with B and N are shown. C1s spectra of LIG samples are further deconvoluted to understand the bonding states for C atoms and changes due to doping. The deconvolution of LIG_U_ has 3 components in which the XPS peak at 284.4 eV [36] is the signature for the main component, graphitic-sp^2^-C=C bond, and similarly at 285.7 eV for the indicative peak of sp^3^-C-C and 287.4 eV for the peak of sp^2_^C=O [29]. Similar observations can be seen from the components of the deconvoluted XPS peak of LIG_B_, along with additional C-O and C-O-B bonding at 285.4 eV [29], which indicates that boron is incorporated into the graphene lattice. Furthermore, the C1s spectrum of LIG_N_ was deconvoluted into 3 essential components that show a characteristic peak of sp^2^-C=N at 288.7 eV and sp^3^-C-N bonding at 285.7 eV. This indicates the affinity of N towards C forming C-N bonds into graphene lattices. In co-doped LIG_BN_, when the XPS peak was deconvoluted, it was divided into 4 components, which suggests that there is a sp^2^-C=C bond at 284.8 eV [29], sp^3^-C-N at 285.7 eV [37], C=O at 286.9 eV, and O-C=O at 289.8 eV.

The insignificant shift of binding energy (C=C from 284.4 eV to 283.6 eV) towards a lower region might be due to a change in the chemical environment of the LIG framework and the presence of oxygen at the ambient condition of the experiment, which is attributed to the doping of B and N [29,38].

To study the bonding states of B with other atoms such as C and N, the XPS survey of LIG_BN_ for B1s was plotted ranging from 184 eV to 196 eV, as shown in Figure 4b. The B1s spectrum for LIG_BN_ shows characteristic peaks that illustrate the successful doping of B into the LIG lattice network [39,40].

Further, moving ahead to the N1s XPS spectra as exhibited in Figure 4c, the spectrum was obtained to determine the different characteristic bonding configurations of N. The presence of characteristics N1s peak of LIG_BN_ demonstrates that N was successfully incorporated into the graphene lattice and caused defect formation [41,42]. Doping boron and nitrogen into the graphene lattices improves the electrocatalytic behaviours and defects of the graphene electrodes, which improves the sensitivity of electrochemical performance for HMs detection [42].

### 3.4. Electrochemical Characterisation

**Figure 5:**
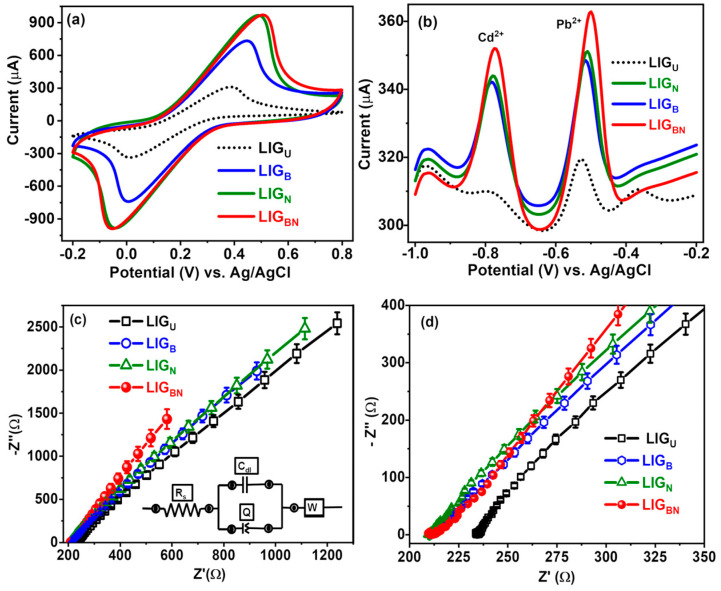
(**a**) Cyclic Voltammograms (CVs) of LIG samples in 5.0 mM [Fe(CN)_6_]^3−/4−^ containing 0.1 M KCl at 50 mV/s, (**b**) square wave voltammograms (SWVs) of LIG samples in 0.1 M NaAc/HAc buffer (pH = 4.5) containing 40 µM Pb^2+^ and Cd^2+^, (**c**) Nyquist plot of LIG samples in 5.0 mM [Fe(CN)_6_]^3−/4−^ containing 0.1 M KCl from 0.1 Hz to 100 kHz (the inset shows the Randle circuit of equivalent resistance), and (**d**) enlarged view of Nyquist plot.

Electrochemical characterisations of LIG_BN_ were first studied by adopting [Fe (CN)_6_]^3−/4−^ as electrochemical redox probes. Figure 5a shows the CVs of LIG_U_, LIG_B_, LIG_N_, and LIG_BN_-modified electrodes in 5.0 mM [Fe (CN)_6_]^3−/4−^ containing 0.1 M KCl. A pair of well-defined reversible redox peaks is exhibited at the CVs of LIG electrodes. Compared with the anodic peak current of LIG_U_ (310.46 μA), there is more than a 3-times increase in the peak current of LIG_BN_ (970.90 μA) due to the doping of B and N and resulting from the synergistic effect of boron and nitrogen, which accelerates the electron transfer rate [29]. Additionally, the electrochemical active surface area (EASA) for LIG_U_ and LIG_BN_ was calculated using the Randle Sevcik equation, I_p_ = 2.69 × 10^5^ A × D^1/2^ n ^3/2^ v ^1/2^ C, where I_p_ is the peak current value, n is the number of electrons transfer in the redox reaction, A is the active area (cm^2^), D is the diffusion coefficient (cm^2^·s^−1^), C is the redox electrolyte concentration (mol·cm^−3^), and v is the scanning rate (mV·s^−1^). In this CV study, n = 1, D = 6.3 × 10^−6^ cm^2^·s^−1^, and C = 5 × 10^−3^ mol·cm^−3^ [32]. Using the peak current values in Figure 5a, the EASA for LIG_U_ and LIG_BN_ were found to be 0.37 cm^2^ and 1.17 cm^2^, respectively. It indicates that the electrochemically active surface area of LIG_BN_ is increased by approximately three times more than that of LIG_U_. It infers that LIG_BN_ is beneficial to the improvement of the electrochemical performance and the adsorption of heavy metal ions.

Furthermore, SWV was performed for trace heavy metal ions (Cd^2+^ and Pb^2+^) determination in 0.1 M NaAc/HAc buffer (pH = 4.5). The voltammograms of 40 µM Cd^2+^ and Pb^2+^ at different doped LIGs are presented in Figure 5b (LIG_U_: short dot black line; LIG_B_: solid blue line; LIG_N_: solid green line; LIG_BN_: solid red line). At the LIG_U_, the smallest peak currents of Cd^2+^ and Pb^2+^ are observed, suggesting that the LIG_U_ has a lower sensitivity towards Cd^2+^ and Pb^2+^. In contrast, at LIG_BN_, the current responses of Cd^2+^ and Pb^2+^ are much higher than LIG_U_, resulting from the large effective specific surface area, which is beneficial to the adsorption of the probe metal ions. Most importantly, in comparison with other electrodes, LIG_BN_ possesses not only the distinguishable and completely separated current peaks at −0.77 V for Cd^2+^ and −0.49 V for Pb^2+^ but also the highest peak current signals, which revealed that LIG_BN_ has a higher affinity as well as a better SWV performance towards Cd^2+^ and Pb^2+^ due to the effect of B and N co-doped.

### 3.5. Electrochemical Impedance Spectroscopy Studies

The interfacial properties of undoped and doped LIG-modified electrodes were evaluated using EIS to determine their electron transfer and ion transport properties. Figure 5c represents the Nyquist plot obtained for LIG_U_ and doped LIG-modified electrodes in 5.0 mM [Fe(CN)_6_]^3−/4−^ (0.1 M KCl supporting electrolyte) within the 0.1–100 kHz frequency range. It indicates that none of the electrodes exhibits a semicircle in the high-frequency region, which is the characteristic of the lower charge-transfer resistance (R_ct_), and a straight line at low frequencies depicts the characteristic of the diffusion of the electro-active species towards the electrode/solution interface (Warburg impedance) [43]. The experimental EIS data of LIG samples were fitted with modified Randles electrical equivalent circuits, presented as an inset of Figure 5c with a goodness factor of 0.001. The LIG_BN_ electrode has a lower R_ct_ (211.0 Ω) compared to undoped LIG_U_ (234.7 Ω), which suggests higher conductivity and implies the fastest ion migration in the case of LIG_BN_ [44]. This is due to the suitable porosity of LIG promoting ion diffusion and transport, as well as enhanced surface wettability after boron and nitrogen co-doping [44]. The enlarged view of the Nyquist plot is shown in Figure 5d, which shows the lower impedance of LIG_BN_ than LIG_U_.

Previous research [45,46,47,48,49] suggests that N-doping may improve the graphene samples’ hydrophilicity, polarity, and electron transfer [50,51], which might be the reason that the green curve of LIG_N_ is higher than the red curve of LIG_BN_ at the initial section of the dependency i.e., the high-frequency region of Nyquist plot. However, B doping may improve the graphene samples’ surface wettability toward the electrolyte, reducing the electrode’s hostility [45,46]. Because of this, it makes sense that the two dopants’ co-effects would increase the surface polarity of LIG_BN_, resulting in a lower impedance than LIG_U_ [47,48,49]. Moreover, the kinetics of the diffusion/transport processes of electrolyte ions in the electrodes are connected to the characteristic Warburg impedance, which corresponds to the linear region in the Nyquist plots [52]. The samples of LIG_N_ and LIG_BN_ have larger low-frequency slopes than LIG_B_ and LIG_U_, as seen in Figure 5c, indicating faster ion diffusion processes.

### 3.6. Optimisation of the Electrochemical Parameters

**Figure 6:**
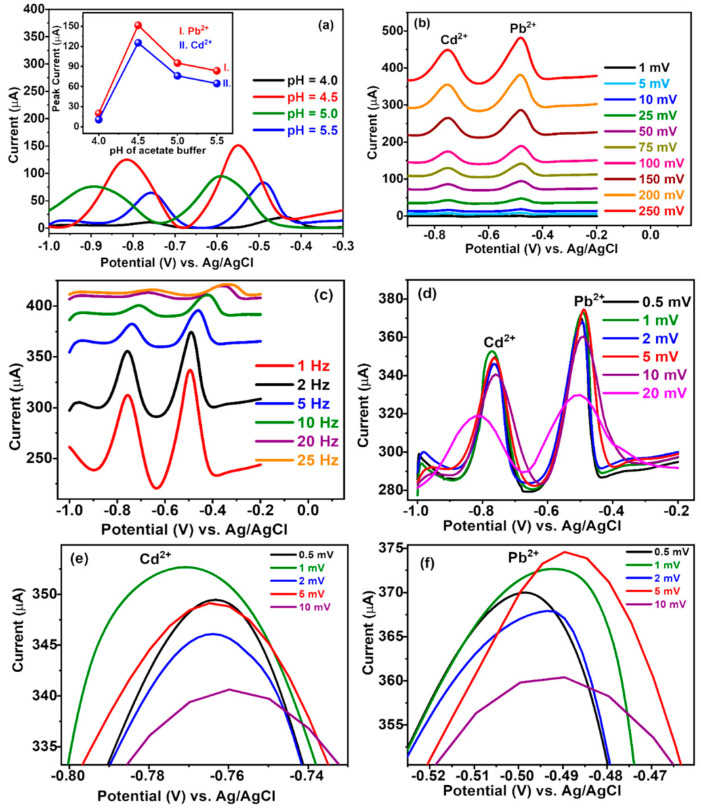
Optimisation of SWV parameters (**a**) pH of 0.1 M NaAc/HAc buffer solution as supporting electrolyte, (**b**) amplitude, (**c**) frequency, and (**d**) potential step (**e**) enlarged plot of SWV peaks on varying potential step for Cd^2+^ and (**f**) enlarged plot of SWV peaks on varying potential step for Pb^2+^.

The experimental conditions affect the heavy metal ions measurement; thus, it is necessary to optimise the measurement conditions to find the best frequency, amplitude, and E-step of the SWV conditions. Firstly, as shown in Figure 6a, we optimised the pH of the 0.1 M NaAc/HAc buffer solution by varying pH from 4.0 to 5.5. The pH of an electrolytic solution depends on the existing state of ions and the stability of the modified electrode. The peak current for both Cd^2+^ and Pb^2+^ is the maximum for pH = 4.5, as indicated by the inset of Figure 6a, which was chosen as the optimal pH of the buffer solution as a supporting electrolyte. The peak current is lower below pH = 4.5, which might be due to the competitive adsorption of H^+^ ions with Pb^2+^ and Cd^2+^ ions onto the electrode surface, which is responsible for the sharp decrease in peak current. Also, the peak current above pH = 4.5 is descended, which might be due to the formation of insoluble hydroxides of heavy metals [53]. The second parameter that needs to be optimised is the amplitude, which was varied in the range of 0 to 250 mV, as shown in Figure 6b, with the frequency fixed at 5 Hz. The peak current gradually increased and the baseline became higher when the amplitude changed from 1 to 250 mV. As the amplitude increased, the peak current increased gradually, and the largest value of the peak current measured was 250 mV. Therefore, the amplitude was selected as 250 mV.

As depicted in Figure 6c, we measured the peak current while changing the frequency from 1 Hz to 25 Hz with the amplitude fixed at 250 mV and the potential step (E-step) at 5 mV. The peak current increased as the frequency increased from 1 Hz to 5 Hz and the SWV flattens after 5 Hz. Therefore, the frequency was selected as 5 Hz.

As shown in Figure 6d, we changed the E-step from 0.5 to 20 mV with the amplitude and frequency fixed. The peak current increased as the E-step increased from 0.5 to 5 mV; yet, after 5 mV, the number of data decreased, and the shape of the SWV curve was not sharp. The broadening of the square wave voltammogram (SWV) with increasing potential steps is due to the diffusion of redox species in the vicinity of the electrode. As the potential step increases, the time required for the redox species to diffuse to the electrode surface also increases. This leads to a broader diffusion layer and a slower rate of mass transfer. Consequently, the current response becomes more spread out over time, resulting in a broader voltammogram [11]. The shift in the maximum of the curve in Figure 6d (20 mV) for cadmium is due to an increase in the step potential. An electrochemical reaction requires a specific lapse of time to ensure the diffusion of the active species and the transfer of charge at the electrode surface, so in case of an increase in potential step, one can limit the time interval of the electrochemical reaction which results in an observed shift towards more negative potentials. The shift is mainly due to the delay of the electrochemical reaction due to the shortness of the allocated time (compared to a lower step potential that allows more time for the reaction to occur) [54]. Figure 6e,f represent the enlarged plot of Figure 6d, showing the separated peaks of Cd^2+^ and Pb^2+^, respectively.

From these results, for the SWV optimal conditions, we determined the pH to be 4.5, the amplitude to be 250 mV, the frequency to be 5 Hz, and the E-step to be 5 mV.

### 3.7. Electrochemical Detection of Pb^2+^ and Cd^2+^ Using SWV

#### 3.7.1. Individual Detection of Pb^2+^ Using SWV

SWV for the individual detection of Pb^2+^ was carried out under optimised experimental conditions.

**Figure 7:**
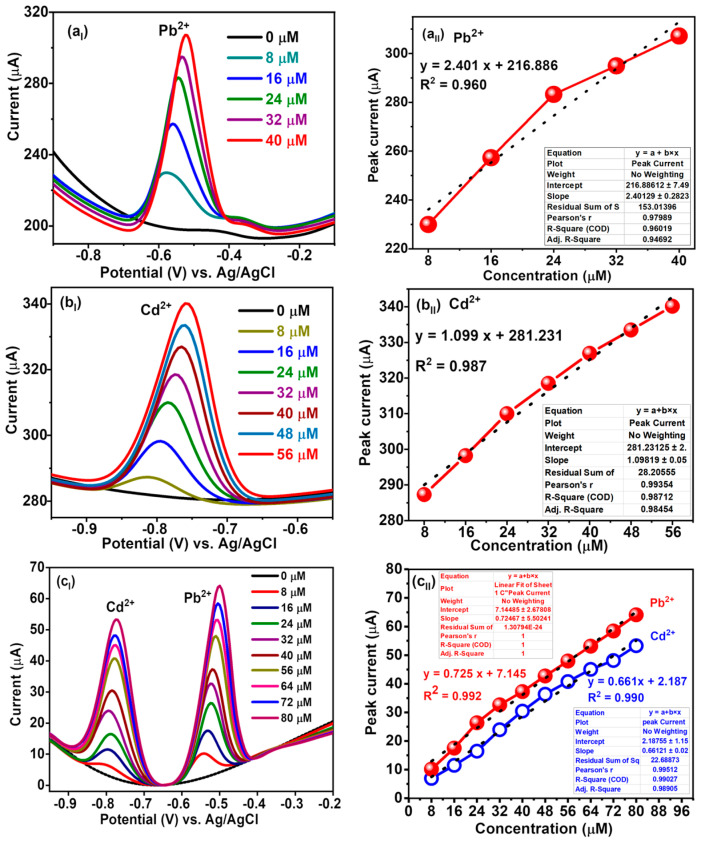
SWV detection of Pb^2+^ and Cd^2+^ in 0.1 M NaAc/HAc buffer pH = 4.5, amplitude = 250 mV, frequency = 5 Hz, and potential step = 5 mV (**a_I_**) SWV response curves for individual detection of 8–40 µM Pb^2+^. (**a_II_**) Calibration curve of SWV peak current versus concentration of Pb^2+^, (**b_I_**) SWV response curves for individual detection of 8–56 µM Cd^2+^; (**b_II_**) calibration curve of SWV peak current versus concentration of Cd^2+^, (**c_I_**) SWV response curves for simultaneous detection of 8–80 µM Pb^2+^ and Cd^2+^, and (**c_II_**) calibration curve of SWV peak current versus concentration of Pb^2+^ and Cd^2+^.

Figure 7a_I_ shows the well-defined peak of Pb^2+^ detected at around −0.52 V. It is obvious that the peak current increased as the concentration of Pb^2+^ increased. The corresponding calibration plot, as shown in Figure 7a_II_, of Pb^2+^ ion shows the linear fit with R^2^ = 0.96 having a wide linear range of 8 µM to 40 µM.

I_p_(µA) = 2.401 C(µM) + 216.886 (1)

where I_p_ is the peak current, C is the Pb^2+^ concentration, and R^2^ is the linear correlation coefficient. It is reported that [55] the limit of detection, LOD = 3 σB/b, in which σB is the standard deviation of the population of the blank responses and b is the slope of the regression line. The LOD was calculated to be 0.25 µM. In addition, the sensitivity is calculated to be 2.401 μA·μM^−1^. Moreover, it can be found that the peak potential of Pb^2+^ changed to a more positive direction with the rise of its concentrations. The shift of the peak potentials of Pb^2+^ is because, with the increase in Pb^2+^ concentrations, Pb^2+^ ions deposited on the electrode surface mainly in the form of multilayer. When a positive scanning potential was applied to the electrode surface, the Pb^2+^ ions reduced on the electrode surface were oxidised to produce an oxidation current, but the Pb^0^ inside could not strip duly, which led to the shift of peak potential [10].

#### 3.7.2. Individual Detection of Cd^2+^ Using SWV

After the experimental optimisation of SWV parameters, the LIG_BN_ sensor was employed to detect Cd^2+^. The SWV curve is shown in Figure 7b_I_ in which the characteristic peak of Cd^2+^ appears at about −0.81 V [56]. As the concentration of Cd^2+^ increases, the peak current value increases as depicted in the calibration plot of Figure 7b_II_, exhibiting a highly linear relationship with the Cd^2+^ concentration in the range of 8 to 56 µM and R^2^ = 0.987.

I_p_(µA) = 1.099 C(µM) + 281.231 (2)

where I_p_ is the peak current, C is the Cd^2+^ concentration, and R^2^ is the linear correlation coefficient. The LOD was calculated to be 0.08 µM. Also, the sensitivity of the LIG_BN_ sensor is 1.099 μA·µM^−1^.

#### 3.7.3. Simultaneous Detection Cd^2+^ and Pb^2+^ Using SWV

SWV was employed to discuss the limit of detection and linear range of the LIG_BN_ for the simultaneous determination of Cd^2+^ and Pb^2+^ under the optimised experimental conditions. Figure 7c_I_ depicts the SWV curves of different concentrations of Cd^2+^ and Pb^2+^ in 0.1 M NaAc/HAc buffer (pH 4.5) at LIG_BN_. The oxidation peaks of Cd^2+^ and Pb^2+^ increase accompanied by the successive increase in metal ions concentration. Well-defined response peaks are observed at around −0.77 V and −0.49 V belonging to Cd^2+^ and Pb^2+^, respectively. Also, the reason for wide linear ranges, as shown in Figure 7c_II_, of 8 µM to 80 µM is due to the porous networked graphene structures of LIG_BN_ due to B and N co-doping, which provide large specific surface areas and abundant reactive sites that facilitate the diffusion of metal ions. Moreover, the LOD is calculated at 0.21 µM and 0.25 µM for Pb^2+^ and Cd^2+^, respectively. In addition, LIG_BN_ requires neither expensive instruments nor complicated and tedious processes. Thus, the promptly fabricated sensor is applicable for sensitive and rapid determination of Cd^2+^ and Pb^2+^ with the merits of simple fabrication, easy operation, and low cost.

The doping effect into LIG, which results in improved electrochemical sensing performance towards HMs, is due to heteroatoms and their synergistic behaviours. Substitutional doping of heteroatoms such as boron (B), nitrogen (N), sulphur (S), phosphorus (P), etc., into the graphene lattices is one of the powerful strategies to surely cause improvements in structural and electronic properties through the creation of sp^3^ defects. Since B has a comparable atomic radius and valence electrons to C, it is considerably simpler to integrate B into a graphene lattice. With B sp^2^ hybridised into the lattice of graphene, the planar geometry of graphene remains preserved. Furthermore, N is easily incorporated into graphene structures due to its comparable atomic size to C and the formation of strong bonds with each other [57]. Also, nitrogen doping into the framework of carbon-based materials has been rapidly progressing to acquire advantageous semiconducting characteristics. The electron-deficient nature of B creates P-type doping, whereas electron-rich N can donate its lone pair electrons, behaving as an N-type dopant. Also, because of the established synergy between the dopants, doping electron-rich and electron-deficient dopants at the same time can result in distinct electrical characteristics. In comparison to the undoped situation, B and N co-doping generates more catalytically active sites, resulting in higher catalytic activity, which improves the adsorption behaviour of heavy metal ions towards doped graphene. Also, it enhances the anchoring of functional moieties or molecules and accelerates charge transfer between electrode and analyte/electrolyte, all of which would boost electrochemical sensing efficiency [58].

Table 2 shows the comparison of developed electrochemical sensors with the present work reported for the simultaneous detection of Pb^2+^ and Cd^2+^ [59,60,61,62,63].

In a nutshell, the superior electrochemical performance of LIG_BN_ is due to the porous graphene nanostructures, as evidenced by Figure 2, and increased sp^3^ defects. The 3D porous interconnected graphene structure increases the electrochemical active specific surface area for diffusion of target ions from the electrolyte onto the electrode [21]. The porosity also enhances the conductivity. Moreover, this phenomenon accelerates electron transfer at interfaces, which enhances the peak current of Pb^2+^ and Cd^2+^ ions. In addition to these, the doping of B and N generates more electrochemical active sites, as proven by an increase in defect density from Raman analysis (Figure 3a). These structural defects are responsible for the creation of active adsorption sites for Pb^2+^ and Cd^2+^ ions, which improves electrochemical sensing performance. The presence of B and N heteroatoms contributes to the structural stability and metal ion affinity. The graphitic N and pyridinic N greatly accelerate electrocatalytic behaviour due to the availability of their lone pair electrons present in them. These inferences result in the fact that LIG_BN_ has the best ability to facilitate electron transfer as well as makes the fastest response to redox reactions. The doping of B and N can create structural defects as also corroborated by Raman studies, which increases the active adsorption sites for the heavy metal ions [54]. Moreover, the electrocatalytic and electronic properties of LIG are improved, which further enhances the electrochemical detection of heavy metal ions [64]. Thus, LIG_BN_ is considered a potential electrochemical sensor platform for the individual and simultaneous detection of Cd^2+^ and Pb^2+^.

## 4. Conclusions

To summarise this study, we propose a facile, novel, and green fabrication route for the B and N co-doped LIG, using simple drop casting and double laser writing technique, which is applied for the electrochemical sensing and detection of Cd^2+^ and Pb^2+^ using SWV. FESEM and HR-TEM analysis showed that porous and interlinked graphene networks are responsible for the enhancement of the specific surface area. Raman studies evidenced that the B and N co-doping introduced defects into the lattice of LIG_BN_. The XPS spectra of LIG samples inferred the successful doping of heteroatoms B and N, showing the bonding configurations at their characteristic binding energies. The electrochemical characterisation of LIG samples carried out by CV, SWV, and EIS study showed that the LIG_BN_ electrode has a higher electrochemical sensing ability than other modified electrodes. After optimising parameters, the LIG_BN_ sensor could respond to individual and simultaneous detection of Cd^2+^ and Pb^2+^ ions. The sensor has a sensitivity of 0.725 µA/µM and 0.661 µA/µM for Pb^2+^ and Cd^2+^ ions, respectively, low limit of detection (0.21 µM and 0.25 µM for Pb^2+^ and Cd^2+^), wide linear ranges (8.0 to 80 µM for Pb^2+^ and Cd^2+^ ions) and high linearity of R^2^ = 0.99 for the simultaneous detection. Overall, this study provides an eco-friendly, simple, and efficient synthesis method for preparing doped electrode sensors. It also sheds new light on the subsequent development of metal-free doped LIG materials for electrochemical detection of Pb^2+^ and Cd^2+^ ions.

## Figures and Tables

**Table 1:** I_D_/I_G_, I_2D_/I_G_, crystallite size, the distance between defects, and defect density of LIG samples.

Samples	I_D_	I_G_	I_2D_	I_D_/I_G_	I_2D_/I_G_	L_D_	nD (×10^11^)	FWHM of 2D	L_a_
LIG_U_	1289.808	3848.702	2882.18	0.33	0.75	23.95	1.00	66.34	149.20
LIG_B_	2432.031	4749.7998	3623.687	0.51	0.76	19.37	1.53	70.70	97.65
LIG_N_	2970.914	4483.142	3272.021	0.66	0.73	17.03	1.99	80.12	75.45
LIG_BN_	6281.095	6513.148	4475.658	0.96	0.69	14.12	2.89	89.56	51.84

**Table 2:** A comparison of the electrochemical sensors reported for simultaneous detection of Pb^2+^ and Cd^2+^.

Electrode	Electrochemical Techniques	Linear Range (µM)		LOD (µM)		Ref.
		Pb^2+^	Cd^2+^	Pb^2+^	Cd^2+^	
^a^PANI/^b^GCE	^k^SWASV	0–2.0	0–2.0	0.10	0.13	[59]
In situ ^c^Sb^d^SPCE	^l^DPASV	0.08–0.30	0.10–0.60	20	30	[60]
P(^e^DPA-co-2^f^ABN)/GCE	^m^DPV	1.25–283.2	11.20–8076	0.7963	2.2684	[61]
Sb^g^F/^h^GO/SPCE	^n^SI-SWASV	0.1–1.3	0.3–1.5	0.026	0.054	[62]
Nafion/^i^CLS/^j^PGR/GCE	DPASV	0.05–5.0	0.05–5.0	0.01	0.003	[63]
LIG_BN_	^o^SWV	8–80	8–80	0.21	0.25	This work

^a^PANI: polyaniline; ^b^GCE; glassy carbon electrode; ^c^Sb: antimony; ^d^SPCE: screen-printed carbon electrode; ^e^DPA: diphenylamine; ^f^ABN: aminobenzonitrile; ^g^F: film; ^h^GO: graphene oxide; ^i^CLS: calcium lignosulphonate; ^j^PGR: porous graphene; ^k^SWASV: square wave anodic stripping voltammetry; ^l^DPASV: differential pulse anodic stripping voltammetry; ^m^DPV: differential pulse voltammetry; ^n^SI-SWASV: sequential injection square wave anodic stripping voltammetry; ^o^SWV: square wave voltammetry.

## Data Availability

Data will be made available on request.

## References

[1] Briffa J., Sinagra E., Blundell R. (2020). Heavy Metal Pollution in the Environment and Their Toxicological Effects on Humans. Heliyon.

[2] Mohammed A.S., Kapri A., Goel R. (2011). Heavy Metal Pollution: Source, Impact, and Remedies. Biomanagement of Metal-Contaminated Soils.

[3] Rama Jyothi N. (2020). Heavy Metal Sources and Their Effects on Human Health.

[4] Tchounwou P.B., Yedjou C.G., Patlolla A.K., Sutton D.J. (2012). Heavy Metal Toxicity and the Environment. EXS.

[5] Wani A.L., Ara A., Usmani J.A. (2015). Lead Toxicity: A Review. Interdiscip. Toxicol..

[6] Genchi G., Sinicropi M.S., Lauria G., Carocci A., Catalano A. (2020). The Effects of Cadmium Toxicity. Int. J. Environ. Res. Public Health.

[7] Trzcinka-Ochocka M., Brodzka R., Janasik B. (2016). Useful and Fast Method for Blood Lead and Cadmium Determination Using ICP-MS and GF-AAS; Validation Parameters. J. Clin. Lab. Anal..

[8] Lan G., Li X., Jia H., Yu X., Wang Z., Yao J., Mao X. (2022). Fast and Sensitive Determination of Cadmium and Selenium in Rice by Direct Sampling Electrothermal Vaporization Inductively Coupled Plasma Mass Spectrometry. Molecules.

[9] Beltrán B., Leal L.O., Ferrer L., Cerdà V. (2015). Determination of Lead by Atomic Fluorescence Spectrometry Using an Automated Extraction/Pre-Concentration Flow System. J. Anal. At. Spectrom..

[10] Zhao G., Wang X., Liu G., Thi Dieu Thuy N. (2022). A Disposable and Flexible Electrochemical Sensor for the Sensitive Detection of Heavy Metals Based on a One-Step Laser-Induced Surface Modification: A New Strategy for the Batch Fabrication of Sensors. Sens. Actuators B Chem..

[11] Mirceski V., Skrzypek S., Stojanov L. (2018). Square-Wave Voltammetry. ChemTexts.

[12] Madhuvilakku R., Yen Y.K., Yan W.M., Huang G.W. (2022). Laser-Scribed Graphene Electrodes Functionalized with Nafion/Fe_3_O_4_ Nanohybrids for the Ultrasensitive Detection of Neurotoxin Drug Clioquinol. ACS Omega.

[13] Zuo Y., Xu J., Zhu X., Duan X., Lu L., Yu Y. (2019). Graphene-Derived Nanomaterials as Recognition Elements for Electrochemical Determination of Heavy Metal Ions: A Review. Microchim. Acta.

[14] Tanwar S., Mathur D. (2021). Graphene-Based Nanocomposites as Sensing Elements for the Electrochemical Detection of Pesticides: A Review. J. Solid State Electrochem..

[15] Singh A., Ahmed A., Sharma A., Arya S. (2022). Graphene and Its Derivatives: Synthesis and Application in the Electrochemical Detection of Analytes in Sweat. Biosensors.

[16] Kumunda C., Adekunle A.S., Mamba B.B., Hlongwa N.W., Nkambule T.T.I. (2021). Electrochemical Detection of Environmental Pollutants Based on Graphene Derivatives: A Review. Front. Mater..

[17] Ye R., James D.K., Tour J.M. (2019). Laser-Induced Graphene: From Discovery to Translation. Adv. Mater..

[18] Ray A., Roth J., Saruhan B. (2022). Laser-Induced Interdigital Structured Graphene Electrodes Based Flexible Micro-Supercapacitor for Efficient Peak Energy Storage. Molecules.

[19] Raza T., Tufail M.K., Ali A., Boakye A., Qi X., Ma Y., Ali A., Qu L., Tian M. (2022). Wearable and Flexible Multifunctional Sensor Based on Laser-Induced Graphene for the Sports Monitoring System. ACS Appl. Mater. Interfaces.

[20] Lopes D.V., Santos N.F., Moura J.P., Fernandes A.J.S., Costa F.M., Kovalevsky A.V. (2023). Design of Laser-Induced Graphene Electrodes for Water Splitting. Int. J. Hydrogen Energy.

[21] Lin X., Lu Z., Dai W., Liu B., Zhang Y., Li J., Ye J. (2018). Laser Engraved Nitrogen-Doped Graphene Sensor for the Simultaneous Determination of Cd(II) and Pb(II). J. Electroanal. Chem..

[22] Jeong S., Yang S., Lee Y.J., Lee S.H. (2023). Laser-Induced Graphene Incorporated with Silver Nanoparticles Applied for Heavy Metal Multi-Detection. J. Mater. Chem. A Mater..

[23] Saisree S., Arya Nair J.S., Karunakaran Yesodha S. (2023). Graphene Quantum Dots Doped with Sulfur and Nitrogen as Versatile Electrochemical Sensors for Heavy Metal Ions Cd(II), Pb(II), and Hg(II). ACS Appl. Nano Mater..

[24] Nandee R., Chowdhury M.A., Shahid A., Hossain N., Rana M. (2022). Band Gap Formation of 2D Materialin Graphene: Future Prospect and Challenges. Results Eng..

[25] Kumar R., Sahoo S., Joanni E., Singh R.K., Maegawa K., Tan W.K., Kawamura G., Kar K.K., Matsuda A. (2020). Heteroatom Doped Graphene Engineering for Energy Storage and Conversion. Mater. Today.

[26] Pumera M. (2014). Heteroatom Modified Graphenes: Electronic and Electrochemical Applications. J. Mater. Chem. C Mater..

[27] Peng Z., Ye R., Mann J.A., Zakhidov D., Li Y., Smalley P.R., Lin J., Tour J.M. (2015). Flexible Boron-Doped Laser-Induced Graphene Microsupercapacitors. ACS Nano.

[28] Han S., Liu C., Li N., Zhang S., Song Y., Chen L., Xi M., Yu X., Wang W., Kong M. (2022). One-Step Fabrication of Nitrogen-Doped Laser-Induced Graphene Derived from Melamine/Polyimide for Enhanced Flexible Supercapacitors. CrystEngComm.

[29] Khandelwal M., Van Tran C., Lee J., Bin In J. (2021). Nitrogen and Boron Co-Doped Densified Laser-Induced Graphene for Supercapacitor Applications. Chem. Eng. J..

[30] Mahanta S.K., Suman S., Ghadei S.K., Balaji U., Sakthivel R., Sankaran K.J. (2023). Direct Fabrication of Metal-Free Graphene Nanohairs/Polyimide Heterojunction for the Highly Efficient Photocatalytic Degradation of Industrial Dyes. Diam. Relat. Mater..

[31] Menon D.M.N., Giardino M., Janner D. (2023). Direct Fabrication of Ultrahydrophobic Laser-Induced Graphene for Strain Sensors. Appl. Sci..

[32] Liu X., Wang X., Li J., Qu M., Kang M., Zhang C. (2023). Nonmodified Laser-Induced Graphene Sensors for Lead-Ion Detection. ACS Appl. Nano Mater..

[33] Samantaray S.S., Sangeetha V., Abinaya S., Ramaprabhu S. (2018). Enhanced Hydrogen Storage Performance in Pd_3_Co Decorated Nitrogen/Boron Doped Graphene Composites. Int. J. Hydrogen Energy.

[34] Wang F., Wang K., Dong X., Mei X., Zhai Z., Zheng B., Lv J., Duan W., Wang W. (2017). Formation of Hierarchical Porous Graphene Films with Defects Using a Nanosecond Laser on Polyimide Sheet. Appl. Surf. Sci..

[35] Wang F., Dong X., Wang K., Duan W., Gao M., Zhai Z., Zhu C., Wang W. (2019). Laser-Induced Nitrogen-Doped Hierarchically Porous Graphene for Advanced Electrochemical Energy Storage. Carbon.

[36] Mannan M.A., Hirano Y., Quitain A.T., Koinuma M., Kida T. (2019). Graphene Oxide to B, N Co-Doped Graphene through Tris-Dimethylaminoborane Complex by Hydrothermal Implantation. Am. J. Mater. Sci..

[37] Matsoso B.J., Ranganathan K., Mutuma B.K., Lerotholi T., Jones G., Coville N.J. (2016). Time-Dependent Evolution of the Nitrogen Configurations in N-Doped Graphene Films. RSC Adv..

[38] Yu Z., Zhang J., Xing C., Hu L., Wang L., Ding M., Zhang H. (2019). High Energy Density Supercapacitor Based on N/B Co-Doped Graphene Nanoarchitectures and Ionic Liquid Electrolyte. Ionics.

[39] Jiang Z., Zhao X., Tian X., Luo L., Fang J., Gao H., Jiang Z.J. (2015). Hydrothermal Synthesis of Boron and Nitrogen Codoped Hollow Graphene Microspheres with Enhanced Electrocatalytic Activity for Oxygen Reduction Reaction. ACS Appl. Mater. Interfaces.

[40] Molina-García M.A., Rees N.V. (2018). “Metal-Free” Electrocatalysis: Quaternary-Doped Graphene and the Alkaline Oxygen Reduction Reaction. Appl. Catal. A Gen..

[41] Chen S., Li P., Xu S., Pan X., Fu Q., Bao X. (2018). Carbon Doping of Hexagonal Boron Nitride Porous Materials toward CO_2_ Capture. J. Mater. Chem. A Mater..

[42] Zhou S., Zang J., Li W., Tian P., Gao H., Song S., Tian X., Wang Y. (2021). B, N Co-Doped Nanocarbon Derived In Situ from Nanoboron Carbide as Electrocatalyst for Oxygen Reduction Reaction. ChemNanoMat.

[43] Coros M., Varodi C., Pogacean F., Gal E., Pruneanu S.M. (2020). Nitrogen-Doped Graphene: The Influence of Doping Level on the Charge-Transfer Resistance and Apparent Heterogeneous Electron Transfer Rate. Sensors.

[44] Bai L., Ge Y., Bai L. (2019). Boron and Nitrogen Co-Doped Porous Carbons Synthesized from Polybenzoxazines for High-Performance Supercapacitors. Coatings.

[45] Niu L., Li Z., Hong W., Sun J., Wang Z., Ma L., Wang J., Yang S. (2013). Pyrolytic Synthesis of Boron-Doped Graphene and Its Application Aselectrode Material for Supercapacitors. Electrochim. Acta.

[46] Sahoo M., Sreena K.P., Vinayan B.P., Ramaprabhu S. (2015). Green Synthesis of Boron Doped Graphene and Its Application as High Performance Anode Material in Li Ion Battery. Mater. Res. Bull..

[47] Shao C., Qiu S., Chu H., Zou Y., Xiang C., Xu F., Sun L. (2018). Nitrogen-Doped Porous Microsphere Carbons Derived from Glucose and Aminourea for High-Performance Supercapacitors. Catal. Today.

[48] Gopalsamy K., Balamurugan J., Thanh T.D., Kim N.H., Lee J.H. (2017). Fabrication of Nitrogen and Sulfur Co-Doped Graphene Nanoribbons with Porous Architecture for High-Performance Supercapacitors. Chem. Eng. J..

[49] Wang Q., Qin B., Zhang X., Xie X., Jin L., Cao Q. (2018). Synthesis of N-Doped Carbon Nanosheets with Controllable Porosity Derived from Bio-Oil for High-Performance Supercapacitors. J. Mater. Chem. A Mater..

[50] Jiang H., Wang Z., Yang Q., Hanif M., Wang Z., Dong L., Dong M. (2018). A Novel MnO_2_/Ti_3_C_2_Tx MXene Nanocomposite as High Performance Electrode Materials for Flexible Supercapacitors. Electrochim. Acta.

[51] Wu J., Sun Y., Yang X., Long G., Zong Y., Li X., Zheng X. (2018). Effect of Graphene Thickness on the Morphology Evolution of Hierarchical NiCoO_2_ Architectures and Their Superior Supercapacitance Performance. Ceram. Int..

[52] Zhu T., Li S., Ren B., Zhang L., Dong L., Tan L. (2019). Plasma-Induced Synthesis of Boron and Nitrogen Co-Doped Reduced Graphene Oxide for Super-Capacitors. J. Mater. Sci..

[53] Huang R., Lv J., Chen J., Zhu Y., Zhu J., Wågberg T., Hu G. (2023). Three-Dimensional Porous High Boron-Nitrogen-Doped Carbon for the Ultrasensitive Electrochemical Detection of Trace Heavy Metals in Food Samples. J. Hazard. Mater..

[54] Bard A.J., Faulkner L.R., White H.S. (2022). Electrochemical Methods: Fundamentals and Applications.

[55] Lin X., Lu Z., Zhang Y., Liu B., Mo G., Li J., Ye J. (2018). A Glassy Carbon Electrode Modified with a Bismuth Film and Laser Etched Graphene for Simultaneous Voltammetric Sensing of Cd(II) and Pb(II). Microchim. Acta.

[56] Lee S., Oh J., Kim D., Piao Y. (2016). A Sensitive Electrochemical Sensor Using an Iron Oxide/Graphene Composite for the Simultaneous Detection of Heavy Metal Ions. Talanta.

[57] Kaushal S., Kaur M., Kaur N., Kumari V., Singh P.P. (2020). Heteroatom-Doped Graphene as Sensing Materials: A Mini Review. RSC Adv..

[58] Varodi C., Pogăcean F., Cioriță A., Pană O., Leoștean C., Cozar B., Radu T., Coroș M., Ștefan-Van Staden R.I., Pruneanu S.M. (2021). Nitrogen and Sulfur Co-Doped Graphene as Efficient Electrode Material for l-Cysteine Detection. Chemosensors.

[59] Wang Z., Liu E., Zhao X. (2011). Glassy Carbon Electrode Modified by Conductive Polyaniline Coating for Determination of Trace Lead and Cadmium Ions in Acetate Buffer Solution. Thin Solid Film..

[60] Sosa V., Barceló C., Serrano N., Ariño C., Díaz-Cruz J.M., Esteban M. (2015). Antimony Film Screen-Printed Carbon Electrode for Stripping Analysis of Cd(II), Pb(II), and Cu(II) in Natural Samples. Anal. Chim. Acta.

[61] Philips M.F., Gopalan A.I., Lee K.P. (2012). Development of a Novel Cyano Group Containing Electrochemically Deposited Polymer Film for Ultrasensitive Simultaneous Detection of Trace Level Cadmium and Lead. J. Hazard. Mater..

[62] Ruengpirasiri P., Punrat E., Chailapakul O., Chuanuwatanakul S. (2017). Graphene Oxide-Modified Electrode Coated with in-Situ Antimony Film for the Simultaneous Determination of Heavy Metals by Sequential Injection-Anodic Stripping Voltammetry. Electroanalysis.

[63] Yu L., Zhang Q., Yang B., Xu Q., Xu Q., Hu X. (2018). Electrochemical Sensor Construction Based on Nafion/Calcium Lignosulphonate Functionalized Porous Graphene Nanocomposite and Its Application for Simultaneous Detection of Trace Pb^2+^ and Cd2^+^. Sens. Actuators B Chem..

[64] Varodi C., Pogăcean F., Coros M., Magerusan L., Stefan-Van Staden R.I., Pruneanu S. (2021). Hydrothermal Synthesis of Nitrogen, Boron Co-doped Graphene with Enhanced Electro-catalytic Activity for Cymoxanil Detection. Sensors.

